# Marine Algal Antioxidants as Potential Vectors for Controlling Viral Diseases

**DOI:** 10.3390/antiox9050392

**Published:** 2020-05-07

**Authors:** Clementina Sansone, Christophe Brunet, Douglas M. Noonan, Adriana Albini

**Affiliations:** 1 Stazione Zoologica Anton Dohrn, Villa Comunale, 80121 Naples, Italy; christophe.brunet@szn.it; 2 Laboratory of Vascular Biology and Angiogenesis, IRCCS MultiMedica, 20138 Milan, Italy; douglas.noonan@uninsubria.it (D.M.N.); albini.adriana@gmail.com (A.A.); 3 Department of Biotechnology and Life Sciences, University of Insubria, 211000 Varese, Italy; 4 School of Medicine and Surgery, University of Milano-Bicocca, 20900 Monza, Italy

**Keywords:** antiviral, antioxidant, marine, algae, COVID-19

## Abstract

As the COVID-19 epidemic expands in the world, and with the previous SARS epidemic, avian flu, Ebola and AIDS serving as a warning, biomedical and biotechnological research has the task to find solutions to counteract viral entry and pathogenesis. A novel approach can come from marine chemodiversity, recognized as a relevant source for developing a future natural “antiviral pharmacy”. Activities of antioxidants against viruses can be exploited to cope with human viral infection, from single individual infections to protection of populations. There is a potentially rich and fruitful reservoir of such compounds thanks to the plethora of bioactive molecules and families present in marine microorganisms. The aim of this communication is to present the state-of-play of what is known on the antiviral activities recognized in (micro)algae, highlighting the different molecules from various algae and their mechanisms of actions, when known. Given the ability of various algal molecules—mainly sulfated polysaccharides—to inhibit viral infection at Stage I (adsorption and invasion of cells), we envisage a need to further investigate the antiviral ability of algae, and their mechanisms of action. Given the advantages of microalgal production compared to other organisms, the opportunity might become reality in a short period of time.

## 1. Introduction

The ongoing global emergency linked to the Covid-19 pandemic [1] teaches us that viral diseases can be dramatic all over the world in the present period of climate, political, social and globalization change. Scientific solutions were not available and society was unprepared, claiming that research activities for the discovery of compounds for the prevention and treatment of severe and acute viral infections are nowadays a priority.

Oxidative stress—a loss in the balance between the production of free radicals including reactive oxygen species (ROS) and antioxidant cell signaling pathways [2]—can be a key factor of the pathogenesis in many acute or chronical human diseases [3]. Dietary intake of exogenous antioxidants in humans has a well-known role in preventing and protecting cellular oxidative stress. They operate diverse mechanisms of action and possess different therapeutic effects. As a result, modern medicine tends to use them in the prevention and treatment of various oxidative-stress-associated diseases [4].

Viral infections are promoted by oxidative processes, the latter acting on replication in infected cells, cell proliferation inhibition and cell apoptosis induction [5]. In patients affected by herpes simplex [6], it was observed that an increase of the ROS-induced membrane phospholipids peroxidation caused dysfunction of vital cellular processes such as membrane transport and mitochondrial respiration [7]. Epstein-Barr Virus (EBV) infections cause an increase in DNA damage and significant ROS accumulation, and, interestingly, treatment with ROS scavengers was able to lower DNA damage in both mitogen-stimulated and EBV-infected cells [8].

In this framework, discovery and screening of antioxidant compounds with antiviral properties is promising since the treatment of viral diseases requires the suppression of viral replication and cell survival promotion. The most known ROS scavenger compounds belong to different chemical families, such as polyphenols (phenolic acids, flavonoids, anthocyanins, lignans and stilbenes), carotenoids (xanthophylls and carotenes), sterols, or vitamins (vitamins B, D, E, and C) [9].

For instance, there is a lot of evidence of the capability of natural antioxidants such as vitamins C and E (ascorbic acid and tocopherol, respectively) or polyphenols (e.g., curcumin) scavenging ROS levels in infected cells, inhibiting proapoptotic factors and thus reestablishing the intracellular equilibrium between the stress-related proteins (such as c-Jun N-terminal kinases- JNK) and promitotic (MAPK) and transcription factors (NF-kB) [10,11,12,13].

The most effective antioxidants are mainly synthetized by photosynthetic organisms, sharing these precious compounds with herbivorous animals through diet, and bioaccumulating along the trophic web [14], as occurs in marine systems [15]. Indeed, marine organisms represent a rich source of antioxidants, in terms of quantity and/or diversity. Vitamins B_12_, C, D, E, peptides, amino acids, chitooligosaccharide derivatives, carotenoids, sulphated polysaccharides, sterols, phlorotannins, phenolic compounds and flavones are examples of marine antioxidant richness [16].

It is not surprising, therefore, that marine pharmacology is increasingly growing. Recently, Mayer et al. [17] reported that 21 studies were published in 2014/2015, focusing on marine antiviral drugs acting against human enterovirus 71, human cytomegalovirus, human immunodeficiency virus type-1 (HIV-1), human herpes simplex virus (HSV), influenza virus, hepatitis B virus, murine norovirus, respiratory syncytial virus (RSV) or sindbis virus. The mechanism of action of these compounds is known for five of them, although for the others it is still undetermined [17].

The aim of the present study is to show the state of the art, exploring the potential antiviral activity of known marine antioxidant compounds. For that, we explore the relationship between oxidative stress and viral infections, looking for solutions through the deciphering of cell signaling pathways that can inhibit virus replication and infections, and the mechanisms of action of potential antiviral molecules. 

## 2. Viral Infections and Oxidative Stress

The global public health emergency of international concern by 30 January 2020 regards the novel coronavirus [18], which is the direct cause of the severe pneumonia (COVID-19), with a high rate of transmission and infectivity. The 85% of critically ill patients with COVID-19 showed leukocytosis, high levels of monocytes and neutrophils, and lymphopenia [19], which is a lack of lymphocytes, with patients dying with comorbidity and high levels of plasma cytokines [20]. Lymphopenia and hypercytokinemia are directly correlated with increased severity, mortality and a dysregulated immunological response [21]. First epidemiological indications reveal that the COVID-19 patients requiring intensive care are older and are more likely to have hypertension, diabetes, cardiovascular or cerebrovascular pathologies [22]. Aging and chronic diseases induce chronic endothelial dysfunction that provokes disassembly of intercellular junctions, endothelial cell death and blood–tissue barrier disruption, along with enhanced leukocyte adhesion and extravasation, which could contribute to explaining the lymphopenia observed in severe COVID-19 patients [23]. Endothelial dysfunction increases oxidative stress and systemic inflammation, glycocalyx degradation and inducing a procoagulant and antifibrinolytic state [24]. In some, especially old patients, with COVID19 infection together with other persistent comorbidity chronic states, these pathways could induce severe respiratory failure [25]. 

COVID-19 infection enters into the elective targets for viral diseases, which are, more generally, epithelial tissues, lung (influenza virus, coronavirus [26]), liver (HCV and HCV [27]) and cervix (HPV [28]), all of which are strongly sensitive to oxidative stress and damages [29]. 

ROS play a fundamental role in the normal functioning of the immune system and in the induction of proliferation of T cells and immunological defense [30]. Oxidative stress represents a key factor in inflammation cell signaling for the regulation of cytokines and growth factors, as well as for immunomodulation and apoptosis [31]. Recent observations have drawn attention to the possibility that interactions between ROS and the human immunodeficiency virus (HIV) may also play a role in the pathogenesis of many other viruses as well [32]. Indeed, the role of oxidative stress has been demonstrated in a number of viral infection diseases [33], where cell pro-oxidative signaling co-occurred with viral infections [34]. 

Viral infection is generally divided into three stages:

- Stage I, which consists of virus adhesion, adsorption, entry and invasion of cells;
- Stage II, or eclipse phase, during which the cell is forced to replicate multiple copies of virus genome;
- Stage III, or maturity and release of virus particles (virions).

Viruses enter the cell through specific receptors and coreceptors using the phagocytosis mechanism. The virus must then break out of the vesicle by which it was taken up in order to gain access to the cytoplasm [35]. The activation of phagocytes induces the release of pro-oxidant cytokines, such as tumor necrosis factor alpha (TNFa), or interleukin-1 and 6, paralleled with a significant iron uptake by the reticuloendothelial system [36]. In turn, the release of TNFa from activated phagocytes induces different intracellular responses. First, it acts on cell mitochondria inducing a pro-oxidant effect through the inhibition of the site of superoxide production (Site II). Second, TNFa induces the release of nuclear transcription factor kappa B(NF-K-B) from the cytoplasmic inhibitor protein IkB, activating at a genomic level the transcription of cellular and/or viral genes. 

Many viruses induce host cell death, by apoptosis or pyroptosis among others [37], probably to enhance replication and expansion into the entire organism [38]. For example, HIV-1 activates pathways causing CD4 T-cell death in infected hosts due to caspase-1-mediated pyroptosis triggered by abortive viral infection. Pyroptosis corresponds to an intensely inflammatory form of programmed cell death in which cytoplasmic contents and proinflammatory cytokines, including IL-1β, are released. This death pathway relies on the two signature events in HIV infection—CD4 T-cell depletion and chronic inflammation—and creates a pathogenic vicious cycle in which dying CD4 T cells release inflammatory signals inducing enlarged cell death [39]. 

Considering this evidence, antioxidant compounds are able to inhibit immune system cells (lymphocytes) through apoptosis and then release proinflammatory cytokines (IL-1, IL-6 and TNFa) that are likely to prevent lymphopenia, hypoferremia and, thus lower the viral replication in patients suffering severe viral diseases [40]. Antioxidants such as vitamin E [41] are able to prevent the inhibition of the site of superoxide production (Site II), while the NF-kB-induced gene transcription can be inhibited by thiol groups [42] belonging to molecules that strongly interact with the antioxidant system [43,44]. Antioxidant therapy might further be related to the inhibition of virally induced cell death by antioxidants that scavenge peroxides, such as N-acetylcysteine and glutathione peroxidase [45]. It was observed that the antioxidant N-acetylcysteine blocks viral production in monocyte cell lines, which are the major reservoir for HIV in infected patients [46]. The epigallocatechin-3-gallate from green tea blocks HIV entry [47] due to its antioxidant properties. 

## 3. Marine Antioxidants and Antiviral Activity 

Therapeutic treatments to inhibit or reduce virus replication in human cells are unfortunately restricted. Known examples are mostly anti-HIV drugs, such as ritonavir, sequinavir, or antiflu, such as lopinavir, abifìgavan, or anti-Ebola reagents. They all can have short-term or long-term adverse effects [48], which makes it necessary to explore new molecular moieties in the perspective of producing new pharmaceutical tools [49]. Many antiviral drugs are synthetic organic chemicals or natural derived products, for example, from secondary metabolites of plants [50]. In the emergent “blue” technology era, there is a growing interest in marine-derived antiviral compounds. Marine organisms represent a rich source of many antioxidants that are promising for the development of drugs for the prevention and treatment of various chronic and acute human diseases [51]. Thus, thousands of compounds from various marine organisms such as algae, bacteria, fungi, marine invertebrates or sponges have been screened and 21 of them have demonstrated antiviral activities [17]. In 2018, Fedoreyev et al. [2] reported that an antioxidant “soup”, containing echinochrome A (pigment of sea urchins), ascorbic acid (vitamin C) and α-Tocopherol (vitamin E) possess in vitro antiviral activity against RNA-containing tick-borne encephalitis virus and DNA-containing herpes simplex virus type 1 [2]. This study also demonstrated that a synergistic effect of antioxidant molecules can be effective against virus infections. 

While the research and development of antiviral compounds are mainly focused on macroalgae [52], the interest in microalgae and cyanobacteria has increased since they are known to produce many antioxidant molecules, which also possess antimicrobial, anticancer and antiviral activities [53]. Moreover, cyanobacteria and microalgae in general are promising since the relatively low—compared to higher plants—requirements for growth make them good candidates for the production of antiviral agents at an industrial scale [54]. The compounds mainly capable of antiviral activities comprise sulfated polysaccharides, phenolic compounds, and organic acids [55].

In recent years, it has been shown that marine sulfated polysaccharides possess a wide range of bioactivities, such as anticoagulant, antioxidant, anti-inflammatory, antiviral, antibacterial, antiproliferative, antitumor, anticomplementary and antiadhesive activities [56]. Interestingly, the antiviral activity exhibited by these compounds is often related to their immunomodulation and anticancer activities [57]. The double effect, anticancer and antiviral, of these natural compounds is mainly related to their capability to inhibit cell proliferation and to activate IFN signaling pathways that inhibit kidney and liver cancer progression [58].

In general, antiviral compounds extracted from (micro)algae are mainly polysaccharides that have been screened against pathogenic human viruses (Table 1). The mechanisms of action of these compounds against viruses are not fully clarified, but the activity seems to be related to their antioxidant power inhibiting the different stages of the viral infection, and interfering with its adhesion, penetration or replication [59]. 

For example, the sulfated polysaccharide isolated from the cyanobacteria *Spirulina platensis*, named *Spirulan*, has exhibited potent antiviral activity against both the herpes simplex virus type 1 (HSV-1) and the human immunodeficiency virus type 1 (HIV-1) [87]. 

The sulfated polysaccharides (SPs) carrageenans from red algae (Rhodophyta) display antiviral effects on several viral agents [60], and are significantly active against HPV [61]. They predominantly act by inhibiting the binding or the internalization of virus into the host cells [62]. The carrageenan isolated from *Gigartina skottsbergii* exert promising antiviral activities towards diverse strains of HSV-1 and HSV-2 during virus attachment stage [63] and against human rhinovirus (HRV) proliferation by preventing the primary phases of virus replication [64]. A recent in vivo study in mice indicated that low molecular weight carrageenans (3, 5, and 10 kDa), as well as acetylated and sulfated derivatives, were active against influenza virus and reduced the HIV activity by depolymerization and sulfation processes [65]. 

Another class of sulfated polysaccharides is represented by galactans, present in the external membranes of red algae [66]. The various structural types of this class of polysaccharides display relevant antiviral activity against several enveloped viruses, such as HSV-1 and HSV-2, DENV, HIV-1 and HIV-2, and hepatitis A virus [67]. The galactan sulfate (GS), isolated from the red alga *Agardhiella tenera*, displays an effective control against HIV-1 and HIV-2, blocking the virus adhesion to the cell as well as the attachment of gp120 on CD4+ T cell receptor to HIV-1 gp120 [68]. Another sulfated galactan isolated from the red seaweed, *Schizymenia binderi,* presented highly selective antiviral effects against HSV types 1 and 2 by the inhibition of the attachment of virus to host cells [69]. The D,L-galactan hybrid C2S-3, extracted from the marine red alga *Cryptonemia crenulata*, blocks the multiplication of DENV-2 in Vero cell line [70]. 

Alginates are polysaccharides widely distributed in brown algae (Phaeophyceae), which have also been particularly attractive for their antiviral activities [71]. In particular, an alginate named 911 exhibited promising activity against HIV-1 decrementing the activity of reverse transcriptase (RTase), discontinuing the virus adsorption, and immunostimulating the host cells [72]. Alternative inhibitory results were also reported against the hepatitis B virus (HBV), where 911 alginate could inhibit the virus replication by suppressing the activity of DNA polymerase [73]. Furthermore, the sulfated polymannuroguluronate (SPMG) is a promising anti-AIDS drug candidate, inhibiting the robust attachment of HIV-1 gp120 protein with CD4 molecules on the surface of T cells [74]. 

Fucans are high-molecular-weight sulfated polysaccharides, present in the intercellular tissues or mucilaginous matrix of brown algae [75]. Beside many bioactivities, such as antiproliferative, antiadhesive properties can protect the cells from viral infections [76]. For instance, the sulfated fucans from the brown seaweeds, *Dictyota mertensii*, *Lobophora variegata*, *Fucus vesiculosus*, and *Spatoglossum schroederi*, could prevent HIV infection via the blocking effect of the activity of reverse transcriptase [77]. This study highlighted the necessity of sulfate and carboxyl groups in the inhibitory viral activity of the polysaccharides. The compound named MC26, a new type of fucose polysaccharide isolated from the marine brown alga, *Sargassum piluliferum* exhibited a strong anti-influenza virus activity [78]. Furthermore, the sulfated fucans extracted from the brown seaweed *Cystoseira indica* showed a promising activity against HSV-1 and HSV-2 [79]. 

It was suggested that these compounds might act against viral infection through the inhibition of virus adsorption [80]. The polysaccharides fucoidan, based on the sulfated L-fucose, possesses various biological activities, among them activities against many RNA and DNA viruses such as HIV, HSV1-2, dengue virus, and cytomegalovirus [81]. Fucoidans exert their antiviral activities by blocking the interaction of viruses with the cells, thus inhibiting viral-induced syncytium formation [82]. This property led to attachment of fucoidan to the F0 protein resulting in a great antiviral potency of fucoidan, e.g., higher than the antiviral drug ribavirin [83]. Last but not least, fucoidans also display bioactivity on the immune system at cellular and humoral level by increasing macrophage phagocytosis [84]. The glucan laminaran, one of the most common polysaccharides in brown algae, exhibits a great antiviral activity and low toxicity in vivo [78]. Laminaran polysaccharides extracted from brown algae are proficient to prevent the activity of HIV by preventing its adsorption on human-derived lymphocytes and by blocking the ability of HIV reverse transcriptase, thus the virus’ proliferation [72].

Three polysaccharides extracted from marine microalgae, naviculan from the diatom *Navicula directa*, and two others (named A1 and A2) from the dinoflagellate *Cochlodinium polykrikoides* also displayed antiviral activities against several enveloped viruses, such as HIV-1, HSV-1 or influenza virus type A (IFV-A) [71]. The sulfated polysaccharide p-KG03 extracted from another dinoflagellate *Gyrodinium impudicum* has been also reported active against the encephalomyocarditis RNAvirus (EMCV) [85], and against several strains of influenza viruses, with efficiency comparable to some existing drugs [86]. 

The common characteristic between the above-described examples of antiviral activity from algal polysaccharide is the presence of sulfated groups in their chemical structure [96]. Although these compounds might act on Stage III of viral infection, selectively inhibiting reverse transcriptase in the case of HIV, thus hampering production of new viral particles after infection [97], the inhibitory effect generally might refer to Stage I of viral infection. This is a crucial aspect to investigate since the antiviral property starts with the interaction between the molecule and the positive charges of the virus or on the cell surface, preventing penetration of the former into the host cells [98]. Indeed, sulfated exopolysaccharides from some marine microalgae have been hypothesized to interfere with Stage I of viral infection [99]. 

Therefore, small molecules, such as sulfated polysaccharides, represent a good challenge in antiviral drug discovery studies, since the actual antiviral pharmaceutics are proteins and act at the Stage II of the infection. Considering the COVID19 disease [100], this virus is very infective due to the high adhesion capacity on the oral cell surface [101] and for the easy entrance ability through the ACE2 receptor on the lung cell surface (Stage I of the viral infection) [102]. 

Less investigated, but with a high antioxidant activity, and therefore as potential antiviral, are the polyphenolic compounds: antioxidants produced by marine algae, such as flavonoids, cinnamic acid, benzoic acid, gallic acid, quercetin [91] and phlorotannins, the latter being found in brown macroalgae [92], or diatoms [93]. Phlorotannins are tannin derivatives exhibiting numerous bioactivities, such as antioxidant, anti-inflammatory [94] antibacterial, and antimatrix metallopeptidases (MMP) activities [95]. Ahn et al. [93] reported that phloroglucinol derivative 8,8′-bieckol inhibited the activity of recombinant RT and protease of HIV-1 in vitro. Another peculiar polyphenol discovered in some diatoms, (*Haslea* sp.) marennine, presented relevant bioactivity and antiviral properties [95].

## 4. Conclusions

Viral infections are often promoted by oxidative processes, favoring replication in infected cells, induction and inhibition of cell proliferation. Antiviral activities of antioxidants acting in the antiviral infection task can be exploited as the sea is a fruitful reservoir of such compounds. Many algal sulfated polysaccharides—several of them being exclusively marine—present strong antiviral activities. Furthermore, tannins, e.g., phlorotannin—exclusive in brown algae—as well as some phycobiliproteins—exclusive in marine algae—exert antiviral activities. To implement natural antiviral tools, there is a priority of: (i) investigating the antiviral activities and mechanisms of action, and (ii) focusing on microalgae, as fast dividing and easily exploitable organisms. The antiviral activity of various microalgae has been demonstrated, although there is a lack of information on many classes. Since microalgae are known to be rich in bioactive molecules—the so-called biofactory cells—and present a high diversity and complementarity of antioxidant compounds, they are the ideal reservoir of a sea-derived antiviral pharmacy. This is a fundamental aspect since synergy between molecules, and combinations of diverse backbones, can be further effective against virus infections, as demonstrated in the development and success of the HAART combination therapy for HIV. The promising results reviewed in this communication suggest and urge for investment and advanced research into the development of knowledge on marine antiviral drugs and to develop a pipeline reaching into the biodiversity and chemodiversity of (micro)algae screening to screen them with an antiviral focus.

## Figures and Tables

**Table 1:** Antiviral antioxidant compounds isolated from marine algae.

Antiviral Compound	Organism	Virus	Mechanism	References
Carrageenan	Red alga, *Gigartina skottsbergii*	Influenza virus, DENV, HSV-1, HSV-2, HPV, HRV, HIV	Inhibition the binding or the internalization of virus into the host cells (Stage I, II, III)	[60,61,62,63,64,65]
Galactan	Red algae, *Callophyllis variegate*, *Agardhiella* *tenera*, *Schizymenia binderi*, *Cryptonemia* *crenulata*	HSV-1, HSV-2, HIV-1, HIV-2, DENV, HAV	Blockage of the virus adhesion and replication into the host cell. (Stage I, III)	[66,67,68,69,70]
Alginate	Brown algae, *Laminaria hyperborea*, *Laminaria* *digitata*, *Laminaria japonica*, *Ascophyllum* *nodosum*, *Macrocystis pyrifera*	HIV, IAV, HBV	Inhibiotion of the inverse transcriptase in RNA virus (Stage III)	[71,72,73,74]
Fucan	Brown algae, *Adenocytis utricularis*, *Undaria* *pinnatifida*, *Stoechospermum marginatum*, *Cystoseira indica*, *Cladosiphon okamuranus*, *Fucus* *Vesiculosus*	HSV-1, HSV-2, HCMV, VSV, Sindbis virus, HIV-1	Inhibition of cell adhesion (Stage I); blockage of the reverse transcriptase (Stage III)	[75,76,77,78,79,80,81,82,83,84]
Laminaran	Brown algae, *Fucus vesiculosus*, *Saccharina* *longicruris*, *Ascophyllum nodosum*	HIV	Blockage of the reverse transcriptase (Stage III)	[72,78]
Naviculan	Diatoms, *Navicula directa*	HSV-1, HSV-2	Inhibition of hyaluronidase (Stage II)	[71]
p-KG03	Dinoflagellates, *Gyrodinium impudicum*	EMCV	Inhibition (or slowing down) of cytopathic effect (Stage II)	[85,86]
Sulfated Polysaccharides A1 and A2	Dinoflagellates, *Cochlodinium polykrikoides*	Influenza A and B viruses, RSV-A, RSV-B, parainfluenza-2, HIV1, HSV	Inhibition of cytopathic effect (Stage II)	[71]
Calcium spirulan	Cyanophytes, *Arthrospira platensis*	HSV-1, measles, mumps, influenza, polio, Coxsackie, HIV-1, HCMV	Inhibition of the attachment, penetration, replication (Stage I, II, III)	[87]
Nostaflan	Cyanophytes, *Nostoc flagelliforme*	HSV-1, HSV-2, influenza A virus, human cytomegalovirus	Inhibition of cell adhesion and penetration (Stage I, II)	[88]
Allophycocyanin	Cryptomonads	Enterovirus 71	Inhibition of cytopathic effect, delay in synthesis of viral RNA (Stage II, III)	[89]
α-, β-Pheophorbide like	Green alga, *Dunaliella primolecta*	HSV-1	Inhibition of the adsorption and invasion (Stage I, II)	[90]
Phlorotannins phloroglucinol derivative 8,8′-bieckol	Brown alga, *Ecklonia cava;* Diatoms	HIV-1	Inhibition of the penetration and reverse trascriptases (Stage I, II)	[16,91,92,93,94]
Marennine	Diatoms, *Haslea* sp.	HSV-1	Inhibition of the invasion and replication (Stage II, III)	[95]
